# High carnivore population density highlights the conservation value of industrialised sites

**DOI:** 10.1038/s41598-018-34936-0

**Published:** 2018-11-08

**Authors:** Daan J. E. Loock, Samual T. Williams, Kevin W. Emslie, Wayne S. Matthews, Lourens H. Swanepoel

**Affiliations:** 1 Centre for Sustainable Agriculture, Faculty of Natural and Agricultural Sciences, University of the Free State, 205 Nelson Mandela Drive, Park West, Bloemfontein, 930 South Africa; 2 Department of Zoology, School of Mathematical & Natural Sciences, University of Venda, Private Bag X5050, Thohoyandou, 0950 South Africa; 3 Department of Anthropology, Durham University, Durham, DH1 3LE United Kingdom; 4 Institute for Globally Distributed Open Research and Education (IGDORE), Hoedspruit, 1380 South Africa; 5 Department of Environmental Sciences, College of Agriculture & Environmental Sciences, University of South Africa, P.O. Box 392 Pretoria, 0003, South Africa

## Abstract

As the environment becomes increasingly altered by human development, the importance of understanding the ways in which wildlife interact with modified landscapes is becoming clear. Areas such as industrial sites are sometimes presumed to have little conservation value, but many of these sites have areas of less disturbed habitats around their core infrastructure, which could provide ideal conditions to support some species, such as mesocarnivores. We conducted the first assessments of the density of serval (*Leptailurus serval*) at the Secunda Synfuels Operations plant, South Africa, using camera trap surveys analysed within a spatially explicit capture recapture framework. We show that servals occurred at densities of 76.20–101.21 animals per 100 km², which are higher than previously recorded densities for this species, presumably due to high abundance of prey and the absence of persecution and/or competitor species. Our findings highlight the significant conservation potential of industrialised sites, and we suggest that such sites could help contribute towards meeting conservation goals.

## Introduction

Over the last few centuries, there have been rapid and intense environmental changes caused by increasing human numbers, technological advances, and industrialisation^1^. Human alterations to the environment have resulted in a decline in biodiversity, and are elevating extinction rates of species at a global scale^2^. Currently, more than 75% of the terrestrial surface is impacted by humans^3,4^. These human activities are affecting biodiversity and ecosystems on various scales as well as modifying existing habitats, creating unique urban environments and novel ecosystems^5–7^. In many cases, biodiversity can be positively related to human population at a regional scale due, for instance, to an enhanced spatial heterogeneity between rural and urban environments, and the introduction of exotic species^8,9^. The influence of these modifications depends on both the scale and the organisms involved^5^.

Even within the most densely populated and intensively used areas, including urban landscapes, humans rarely utilise all land, and tend to retain significant green or unused areas. These “green spaces”, or unused areas, can hold ecological potential, and can reduce biodiversity loss by managing habitats to support endangered species^10^. However, further research is necessary to understand the impacts of land transformation processes^11^, including unpredicted changes in species communities, posing new challenges to conservation and resource management^12^.

One species that could be impacted by development is the serval (*Leptailurus serval*). The serval is a medium-sized carnivore that feeds primarily on rodents^13^, and is dependent on wetland habitats^14^ that are being rapidly lost globally^15^. The species is listed as Least Concern on the global IUCN Red List of threatened species^16^, but is considered Near Threatened in South Africa^17^. Serval have declined throughout their range^18^, and the principal threats to the species are loss and degradation of their wetland habitat^19^, trade of their skins^20^, and persecution in response to perceived predation of poultry^21^, although they only rarely prey on livestock^16^. Like many other felids, serval maintain stable home ranges where males typically have larger ranges than females^22,23^. While various factors (e.g. resource availability and physical attributes^24^) affect carnivore home range size, for serval the availability of wetland habitats seems to be a key factor^25^. Data on species ecology are critical to planning wildlife management and implementing conservation initiatives^26^, but there have been few studies on serval ecology, and conservation initiatives are hindered by poor knowledge of abundance^18^. This is especially true for the coal-rich wetland habitats of the eastern grassland areas of South Africa where rapid land transformation has occurred due to open cast mining^27^. Because of its reliance on and apparent sensitivity to changes in its preferred wetland habitat, the serval might be an ideal model species through which to investigate the effects of large-scale industrialisation on wetland and riparian areas^25^.

**Figure 1:**
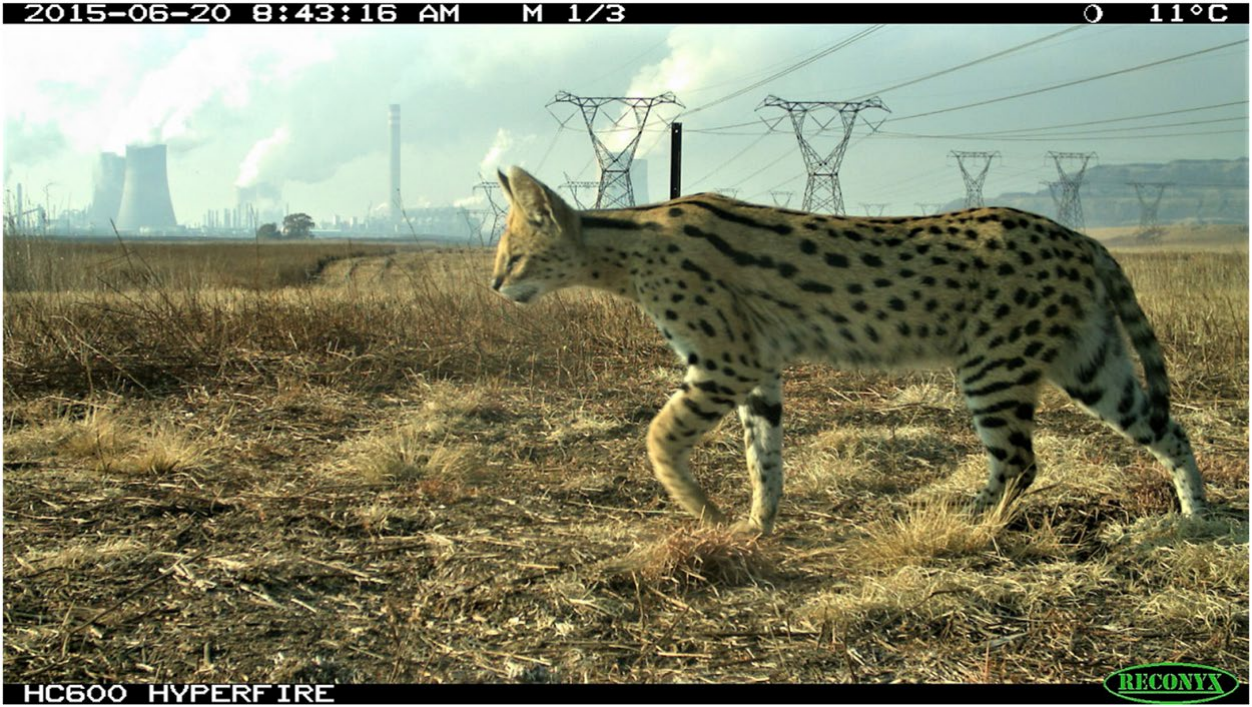
Camera trap image of a serval at the heavily industrialised Secunda Synfuels Operations plant in South Africa, recorded by Reconyx Hyperfire HC600 camera.

In this study, we used the serval as a model species to investigate whether industrial landscapes with varying levels of land and wetland modification can sustain viable populations of specialist carnivores. To achieve this aim, we firstly estimated the population density of servals at the Secunda Synfuels Operations plant, an industrial site in Mpumalanga province, South Africa, which includes a natural wetland within its boundaries (Fig. 1). We then supplemented the estimated densities with live trapping data to assess the structure of this serval population.

## Results

### Camera trapping

During a camera trapping effort of 3,590 trap days, we photographed a total 61 unique serval individuals spanning three separate sessions (Table 1). The number of individual serval captures did not differ greatly between sessions, although the highest number was captured during the wet season of 2015 (Table 1, Fig. S1).

**Table 1:** Summary of camera trapping effort at the study site during the winter of 2014, summer of 2015 and winter of 2015.

Session	Number of days	Number of trap sites	Polygon size (km²)	Photos identifiable	Number of adult serval identified	Captures	Recaptures
2014 Winter	40	34	79.4	332	19	57	32
2015 Summer	40	34	79.4	580	34	87	41
2015 Winter	40	34	79.4	672	31	82	48
Mean	40	34	79.4	528	28	75	40
Total	120	34	79.4	1584	84	226	121

**Figure 2:**
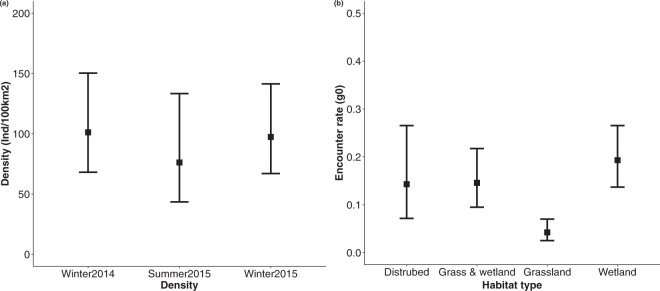
Serval density estimates for each camera trap survey conducted at the study site indicating (**a**) influence of season on density, and (**b**) effect of habitat type on serval encounter rate. Error bars represent asymmetric 95% confidence intervals.

The two most parsimonious SECR models (ΔAICc < 2) both indicated that the encounter rate (${\lambda }_{{{\rm{0}}}_{{\rm{0}}}}$) was affected by habitat type (Table S2). While there was some support for serval density being session dependant (ΔAICc = 0.098; *AICc w* = 0.487; Table S2), there was also support for no effect of session (*AICc w* = 0.471, Table S2). To estimate serval density, we therefore averaged the two most parsimonious models (ΔAICc < 2). Serval population density estimates at the study site varied from 76.20 (SE = 22.22) to 101.21 (SE = 20.66) animals per 100 km² (Fig. 2a). Highest estimates were recorded during the dry seasons (Winter 2014: 101.21 [SE = 20.66] & Winter 2015: 97.38 [SE = 18.71]) compared to the single summer season (Summer 2015: 76.20 [SE = 22.21]; Fig. 2a). Vegetation type had a significant effect on serval encounter rates, where grassland had the lowest encounter rate (0.04 [SE = 0.01]) compared to wetlands with the highest (0.19 [SE = 0.03]; Fig. 2b).

### Live trapping

**Figure 3:**
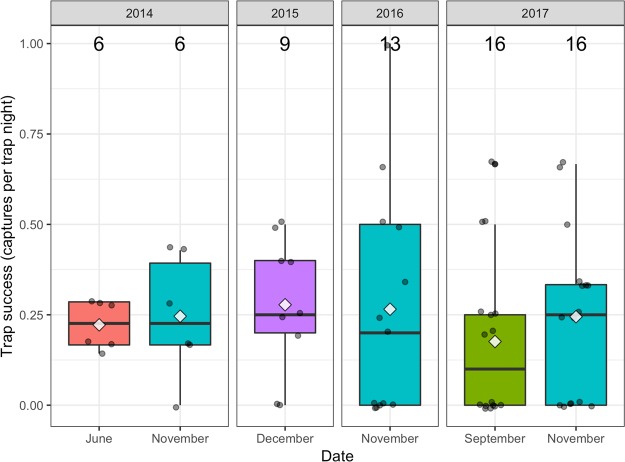
Box plot showing trap success rate for serval captures at the study site from 2014 to 2017. The middle bars represent the median value, white diamonds represent means, the top and bottom of the boxes represent the 75th and 25th percentiles respectively, the whiskers represent the maximum and minimum values, circles show the individual data points, and numbers give the sample size.

We captured 65 individuals, of which four were recaptured on a second occasion. This comprised of a total of 26 adult males, 19 adult females, 11 sub-adults, and seven juvenile animals. This resulted in a mean trapping success rate of 0.21 captures per trap night (excluding recaptures). Trapping success rate varied little between sessions (Fig. 3).

## Discussion

### Comparative serval density

In our three camera trap surveys at the study site at Secunda, we estimated serval population density to be 101.21, 76.20, and 97.38 animals per 100 km², which are the highest densities recorded in the literature. Live trapping rates at Secunda were also extremely high (0.21 captures per trap night). Although there are no data available for serval live trapping rates in the literature, rates of 0.0015–0.0170 captures per trap night are much more typical for other mesocarnivores such as jaguarundi (*Puma yagouaroundi*), oncilla (*Leopardus tigrinus*), tayra (*Eira barbara*), and feral cat (*Felis silvestris catus*) using cage traps^28–30^, which are an order of magnitude lower than serval live capture rates at Secunda. We highlight that our live trapping was biased to prime serval habitat (wetland and disturbed wetlands) and thus, care must be taken when comparing trapping rates between different locations and species. Nonetheless, the live trap rates at Secunda appear to be consistently very high, which supports the high population densities estimated using camera trap data.

Our high estimates of serval densities at Secunda contrast with more typical densities reported in Luambe National Park in Zambia (9.9 animals per 100 km^2^ ^19^), Bwindi Impenetrable National Park in Uganda (9 animals per 100 km^2^ ^20^), and on farmland in the Drakensberg Midlands, South Africa (6.5 animals per 100 km^2 ^^18^). However, there is evidence that serval can attain such high densities. For example, serval densities can reach 41.66 animals per 100 km² in the Ngorongoro Crater, Tanzania^31^.

High population densities of other carnivore species have also been reported in human-modified habitats such as urban areas. Coyotes (*Canis latrans*), raccoons (*Procyon lotor*), red foxes (*Vulpes vulpes*), and Eurasian badgers (*Meles meles*), for example, all thrive in urban landscapes^32,33^. Carnivore species able to adapt to urban environments often succeed in these areas due to high food availability, favourable climatic effects, and the reduced threat of intraguild predation because of the absence of larger apex predators^34^. We provide several, not necessarily mutually exclusive, theories to explain the high serval density we observed at Secunda.

Firstly, servals at Secunda are protected from persecution. Such persecution can have large effects on carnivore densities. For example leopards (*Panthera pardus*) in livestock/game farming areas only attain around 20% of their potential density compared to protected areas free from persecution^35^. Servals outside protected areas are frequently persecuted by livestock farmers^21^ as they are often mistakenly blamed for livestock predation^36^, but at Secunda this is not the case, which could lead to higher population densities^37^. Secondly, servals are the largest remaining carnivore species occurring at ecologically effective densities at Secunda, so there is little interspecific competition from larger carnivores. In other areas, the presence of other medium- and large-bodied carnivores could otherwise limit serval population densities (through intraguild predation), so their absence can lead to mesopredator release, such as through increased survival of young^38^. For example, the absence of large carnivores such as African lions (*Panthera leo*) and spotted hyaenas (*Crocuta crocuta*) in northern South Africa is thought to have led to the competitive release of cheetahs (*Acinonyx jubatus*)^39^. Thirdly, the abundance of modified habitat at Secunda could also facilitate high serval population density. Disturbed habitat can be highly productive^40^ and provide shelter and food resources for species such as rodents that serval prey upon^41^, providing abundant food and in turn supporting a high abundance of serval.

Although the population density of serval recorded at Secunda was exceptionally high, the structure of this serval population was similar to those at other sites. The number of adult males per 100 adult females captured in live traps at Secunda was 137, which is within the range reported in the literature (50–220 in KwaZulu-Natal^25,42^; 100 in the Ngorongoro Crater, Tanzania^31^). Similarly, the proportion of the population at Secunda that was comprised of juvenile and sub-adult individuals (0.69) was very similar to other populations (0.64 in the Ngorongoro crater^31^). It therefore appears that although the serval population density at Secunda is very high, the structure of the population is not unusual, which is not indicative of a rapidly declining or increasing population size^43^, supporting our findings that the serval population density at Secunda appears to be relatively stable. Although servals appear to thrive in close proximity to such a heavily industrialised site, we suggest that further research is conducted to identify any potential effects of industrial activity^44^, such as the influence of noise and air pollution on the physiology and behaviour of wildlife in the vicinity^45^.

While we aimed to apply robust modelling, we address some caveats form our dataset. First, we were not able to include sex as a covariate in the SECR models, which could affect the density estimates. Simulation models suggest that excluding sex covariates can cause a negative bias in density estimates, thus overestimating density^46^. As such it seems that estimates derived here can be regarded as optimistic. Nonetheless, the scale parameter used in the models (sigma = 268 m) falls within range of recommended trap spacing for robust SECR estimates (sigma must be at least 0.5 the average camera spacing^47^). This suggests that the estimated 95% confidence interval should encompass the true estimates, albeit on the lower side of the interval. Secondly, the placement of the camera traps was constrained by the vegetation conditions in order to enable access to camera traps by foot or by vehicle. This could have introduced sampling bias as traps were not placed at random in relation to activity centres. However, maximising the detection of individuals in order to obtain adequate samples outweighs the potential bias caused by biased trap placement^46,47^. Finally, there might be concern regarding population closure since our trapping period spanned 40 days and we had a high percentage of single detections. We highlight that SECR models appear to be robust against transience^48^ and that longer surveys tend to yield more robust estimates than short periods^49^.

### The impacts of modified landscapes

In recent years the expansion of infrastructure has progressed more rapidly than during any other period in history^50^, and industrial sites such as mines and fossil fuel processing plants are not the only developments that could have impacts on wildlife. The growing road network, for example^51^, has large direct and indirect ecological impacts such as causing wildlife-vehicle collisions^52,53^, polluting the environment, disrupting animal migrations and gene flow, and providing access to invading species and humans, facilitating further degradation^54,55^. The rapidly growing number of hydroelectric dams^56^ increases the risk of habitat fragmentation through deforestation, in addition to disrupting freshwater ecosystems^57^. Similarly, the development of urban and agricultural areas fragments and destroys habitats^58^. Consequently, delineating how the changing environment affects biodiversity will be an increasingly important theme of future research.

But not all the impacts of anthropogenic development on wildlife are negative. The high serval densities at Secunda are remarkable as the site is very heavily industrialised. Nature reserves and exclusion zones surrounding industrialised areas such as Secunda have the potential to balance resource utilisation with biodiversity conservation^59^. Some industrial installations such as mines have created nature reserves, which can benefit biodiversity conservation. The Mbalam iron ore mine in Cameroon has set aside land to protect rare forest mammals^59^. Private nature reserves created around the Venetia diamond mine in South Africa and the Jwaneng diamond mine in Botswana support a broad complement of large mammals including elephants (*Loxodonta africana*), African lions, leopards, cheetahs, African wild dogs (*Lycaon pictus*), brown hyaenas (*Hyaena brunnea*), and black-backed jackals (*Canis mesomelas*)^10,60–62^. The Sperrgebiet exclusion zone in Namibia, established to protect diamond deposits^59^, has now been proclaimed a National Park^63^. The consequent changes in the ecological functions of these human modified areas can produce a new combination of species, sometimes modifying and, in some cases, increasing the local richness^7,64^.

Studies such as this highlight the complexity of the relationship between wildlife and the human-modified environment, and suggest that the potential conservation value of industrialised sites should not be overlooked. This underscores the importance of sound ecological management in these areas. Such sites could be incorporated into wildlife management plans, and could help to achieve goals such as the conservation of threatened species. This could be achieved, for example, through the formation of partnerships between industry and the non-profit sector or governmental agencies, such as the partnership between Eskom and the Endangered Wildlife Trust (EWT) to reduce the threats posed by electricity infrastructure to wildlife in South Africa^65^.

## Conclusion

Servals occur at much greater densities at Secunda than have been recorded elsewhere. Capture rates on both camera traps and live traps were remarkably high. High densities may be due to favourable conditions such as a high abundance of rodent prey and the absence of persecution or competitor species. Despite the highly industrialised nature of the site, serval population structure appears to be similar to other natural sites. We suggest that the potential value of industrial sites, where they include areas of relatively natural habitats, may be underappreciated by conservationists, and that these sites could help meet conservation objectives.

## Materials and Methods

### Study site

**Figure 4:**
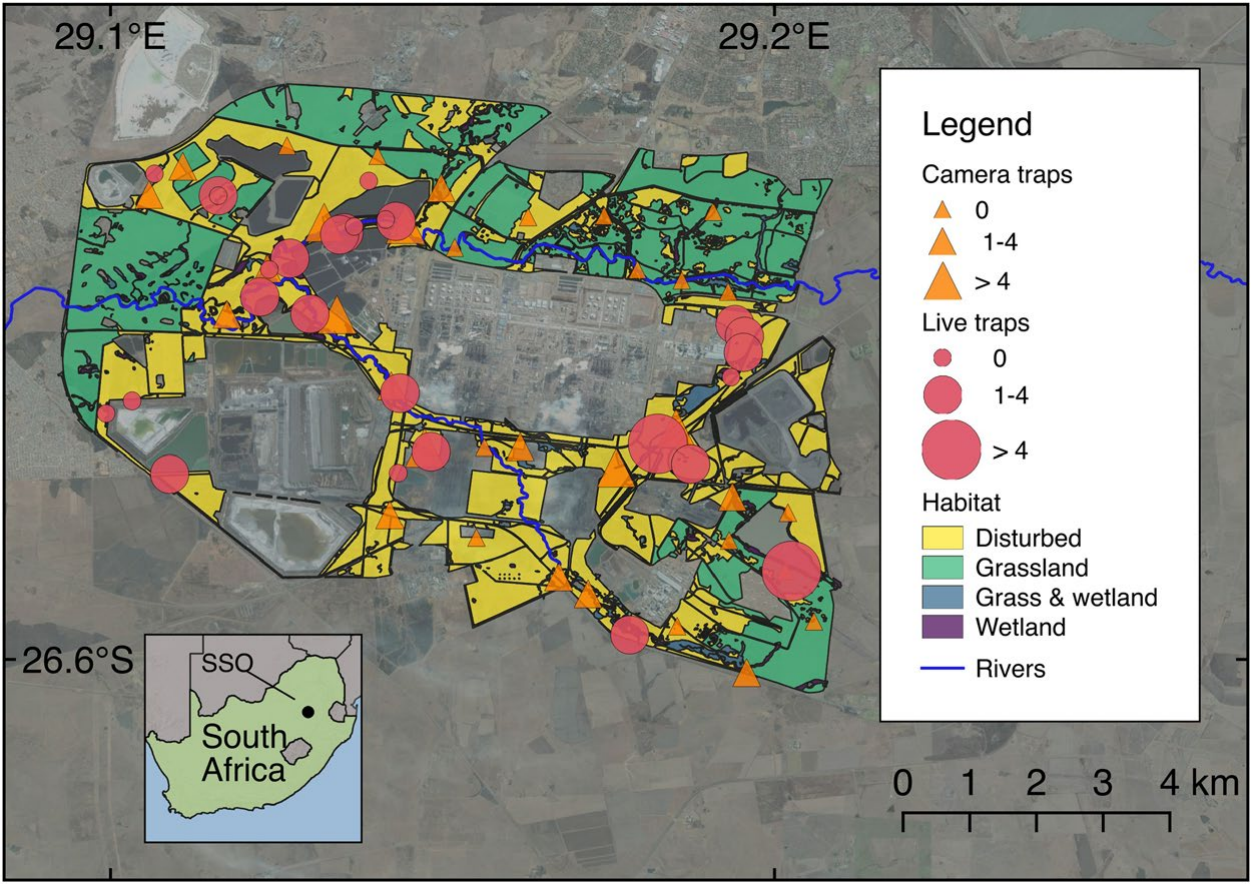
Map showing the locations of camera traps and live traps at the Secunda Synfuels Operations plant in South Africa. The size of points representing camera traps and diameter of live traps are proportional to the number of individual serval captured. Major habitat types are also shown, along with satellite images illustrating the human-modified landscapes. Wetland and Grass & wetland habitat types are difficult to visualise at this scale as they occur in very close proximity to rivers. The figure was created using QGIS 3.0.2^82^ (http://qgis.osgeo.org), and it contains modified Copernicus Sentinel data (satellite image)^67^. QGIS software is released under a GNU General Public License.

The Secunda Synfuels Operations plant (hereafter referred to as Secunda) is a division of Sasol South Africa (PTY) Ltd, and is located in Secunda, Mpumalanga province, South Africa (Fig. 4). It consists of a primary area (a petrochemical plant) and a secondary area (which is made up of surrounding natural and disturbed vegetation). The secondary area (hereafter referred to as the study site) of Secunda Synfuels Operations covers an area of 79.4 km² (central coordinates 26°31′45.62′′ S, 29°10′31.55′′ E). The secondary area is a gently to moderately undulating landscape on the Highveld plateau, supporting short to medium-high, dense, tufted grasses at different levels of disturbance. In places, small scattered wetlands (both man-made and natural), narrow stream alluvia, and occasional ridges or rocky outcrops interrupt the continuous grassland cover. Much of the study site (38%) is classified as relatively untransformed habitat, which is managed in accordance to Secunda Synfuels Operations Biodiversity Management Plan to conserve the natural areas from degradation and improve the ecological functionality of the disturbed land. The vegetation type is classified as Soweto Highveld Grassland^66^, and the area falls within the Grassland Biome^66^. We used satellite images^67^ and existing habitat maps^68^ to digitise the boundaries of four major habitat types (Disturbed, Grassland, Grass & wetland, and Wetland), which we used as site covariates in subsequent analyses.

The relatively unspoiled grassland represents the best form of Soweto Highveld Grassland on site. The characteristic species include *Cymbopogon pospischilii*, *Pollichia campestris*, *Walafrida densiflora, Eragrostis chloromelas*, *Gomphrena celosioides*, *Craibia affinis* and *Cineraria cf. savifraga*^68^. The grassland habitat has a low basal cover due to grazing and during the rainy season the grass phytomass averages around 3–4 tons per hectare^69^. The grass and wetland habitat occurs mostly within the transition zones or dry floodplains not typical of either wetland habitat or grassland habitat. These areas have a medium cover, and include some species typical of wetlands. The wetland habitat is dominated by species indicative of wetland zones and moist soils^70^. The disturbed habitat is dominated by weedy forbs with medium to very high density.

### Camera trapping

The study was underpinned by a spatially explicit capture-recapture (SECR) framework^46,47^. Key recommendations for SECR studies are that the camera trapping polygon be larger than the male home range size of the target species^46^ and that camera placement maximises spatial captures and recaptures^47^. To adhere to these recommendations, we first subdivided the study area into 34 grid cells measuring 1.2 × 1.2 km (roughly equivalent to the size of smallest recorded serval home range^13^). We chose the smallest home range since the deployment of camera traps in such a small area will guarantee that all other age-sex class will be exposed to an equal or larger number of traps^71^. We then established an array of Reconyx Hyperfire HC600 camera traps at 34 camera trap stations (one in each grid cell) over an area of 79.4 km² throughout the study site (Fig. 4). This camera trapping polygon was larger than the largest home range recorded for serval in South Africa (measured using minimum convex polygon) of 31.5 km^2 ^^25^. Mean spacing between camera traps was 885 m. We placed camera traps on game trails and roads to maximise the probability of photographing servals^71^, and to facilitate access for camera maintenance. We visually described the dominant habitat type (based on habitat maps^68^) for a radius of 100 m around each camera trap (classified as Disturbed, Grassland, Grass & wetland, and Wetland), which was used as co-variates in subsequent modelling of detection probability. We mounted camera traps on fence posts, 50 cm above the ground and 1 to 2 m from the trail. Vegetation in front of the camera traps was cleared to reduce false triggers.

We conducted three surveys from 2014 to 2015, with each survey running for 40 days (see Table 1 for dates). Camera traps were programmed to operate 24 hours per day, with a one minute delay between detections. We regarded each 24-hour period as an independent sample. Camera trap positions were kept constant within each survey and between surveys. We visited each camera trap on a weekly basis to download the images, change batteries, and ensure the cameras remained in working order. Camera Base 1.4^72^ was used to catalogue the camera trap images. Since one of the assumptions of SECR models is that individuals are correctly identified, three authors (DL, WM, KE) identified individual serval in triplicate using distinct individual markings such as spot patterns and scars.

### Live trapping

Live trapping formed part of a larger study investigating serval spatial and disease ecology. Here, we used the live trapping data to estimate the capture rate and population structure of the serval population to validate our camera trapping study. Serval were trapped using 16 steel trap cages measuring 200 cm × 80 cm × 80 cm, deployed at 29 trap sites throughout the study site. Traps were baited with dead helmeted guineafowl (*Numida meleagris*) for a total of 287 trap nights between 2014 and 2017. Servals were immobilised by a veterinarian using one of the following drug combinations, as part of a study into optimising immobilisation protocols: (1) KBM-5: ketamine (5.0 mg kg^−1^), butorphanol (0.2 mg kg^−1^), and medetomidine (0.08 mg kg^−1^); (2) KBM-8: ketamine (8.0 mg kg^−1^), butorphanol (0.2 mg kg^−1^), and medetomidine (0.08 mg kg^−1^); (3) ZM: zoletil (5.0 mg kg^−1^) and medetomidine (0.065 mg kg^−1^); (4) AM: alfaxalone (0.5 mg kg^−1^) and medetomidine (0.05 mg kg^−1^); or (5) ABM: alfaxalone (2.0 mg kg^−1^), butorphanol (0.2 mg kg^−1^), and medetomidine (0.08 mg kg^−1^). Drugs were administered intramuscularly using a blowpipe. If serval showed signs of inadequate drug dosages, they were topped-up with the same combinations. Where administered, medetomidine and butorphanol were pharmacologically antagonised with atipamezole (5 mg mg^−1^ medetomidine) and naltrexone (2 mg mg^−1^ butorphanol), respectively. After examination, animals were released at the same site where they were captured.

Animals with a mass of 3–8 kg were considered to be juveniles (up to approximately six months old, to the stage where the canines are developed). Servals with a mass of 8–11 kg were categorised as sub-adults (6–12 months old, just before they are sexually mature). Animals 11–15 kg (approximately 12 to 18 months and older) were considered to be adults^22^.

All methods were carried out in accordance with relevant guidelines and regulations relating to wildlife research in South Africa. All protocols were approved by the Animal Care and Use Committee of the University of Pretoria (Ethical Clearance Number: EC040-14 and V101-17) and the Mpumalanga Tourism and Parks Agency (Permit Number: 5467 and 7282).

### Data analysis

We estimated serval density by fitting likelihood based SECR^73^ models to camera trap data using the package secr^74^ in R version 3.4.3^75^. The advantage of SECR models over traditional density estimation methods is that they do not require the use of subjective effective trapping areas, and instead estimate density directly^46^. This is achieved by estimating the potential animal activity centres in a predefined area using spatial location data from the camera traps^73^. The spacing of the activity centres is related to the home range size of the animals, and as such the detection probability of each animal is a function of the distance from the camera trap to the activity centre. A key assumption of SECR models is that such activity centres are stationary for the period of study (closed population^48^). Since serval are long lived animals exhibiting territoriality, and we had a relatively short survey period we believe that our study did not violate this assumption^31,76^.

Detection rate was modelled using a spatial detection function which is governed by two parameters; the encounter rate at the activity centre (detection probability; *λ*_0_) and a scale parameter ($\sigma$) which describes how the encounter rate declines with increased distance from the activity centre^73^. We tested for three different spatial detection functions since these might better model the utilisation distribution of the home range: half-normal, hazard and exponential. We ranked models based on Akaike information criterion corrected for small sample sizes (AICc), and found overwhelming support for the hazard rate spatial detection function (Table S2). All subsequent models were fitted with the hazard rate detection function.

We fitted SECR models by maximising the full likelihood where the scale parameter was kept constant, but we let the encounter rate vary by biologically plausible hypotheses to deal with heterogeneity in detection. The scale parameter is largely affected by home range size, and hence the sex of the animal^77^. However, we were unable to determine the sex of individual serval from the photographs, and could therefore not model variation in the scale parameter due to sex. We first fitted a model in which we allowed the scale parameter to vary by year and season. This is because we expected that movement might be constrained in the wet season due to increased food resources^78^. We then fitted a model in which serval showed a behavioural response at ${\lambda }_{0}$, as animals can become trap happy or trap shy^79^. Thirdly, we tested the effect of habitat on ${\lambda }_{0}$, as serval prefer wetlands^25^, which would result in higher detections in these habitats. We captured camera-specific habitat variables from the vegetation classification. Fourth, we coded each year and season as a separate session, and used the multi-session framework in secr to test the effect of season on serval density, with constant ${\lambda }_{0}$. We lastly fitted a model in which ${\lambda }_{0}$ varied with both season and habitat type. These models were contrasted against a null model, in which all variables were kept constant.

We used AICc to rank models, considering models with ΔAICc < 2 to have equal support. We applied model averaging to the top models with equal support to reduce uncertainty^80^. The buffer width for analysis was set at 3,000 m, which resulted in the inclusion of an informal housing settlement and a residential area in the state space buffer. Since it is highly unlikely that serval will utilise these areas (as well as the primary industrial area), we excluded these areas (constituting approximately 25% of the area of the buffer) from the state space buffer. All data and R code used for analysis are publicly available^81^.

## Electronic supplementary material

Supplementary information
