# Supplementary material for: High carnivore population density highlights the conservation value of industrialised sites
Source: Sci Rep. 2018 Nov 8;8:16575. doi: 10.1038/s41598-018-34936-0 (PMC6224568; doi:10.1038/s41598-018-34936-0)
Supplement: Supplementary file 1 — Supplementary information [file 41598_2018_34936_MOESM1_ESM.pdf]

Supplementary information

High carnivore population density highlights the conservation value of industrialised sites

Daan J. E. Loock[a*], Samual T. Williams[b,c,d*,] Kevin W. Emslie[b], Wayne S. Matthews[e], Lourens H. Swanepoel[b]

[a] Centre for Sustainable Agriculture, Faculty of Natural and Agricultural Sciences, University of the Free State, 205 Nelson Mandela Drive, Park West, Bloemfontein, 930, South Africa

[b]Department of Zoology, School of Mathematical & Natural Sciences, University of Venda, Private Bag X5050, Thohoyandou, 0950, South Africa

[c]Department of Anthropology, Durham University, Durham, DH1 3LE, United Kingdom

dInstitute for Globally Distributed Open Research and Education (IGDORE)

[e]Department of Environmental Sciences, College of Agriculture & Environmental Sciences, University of South Africa, P.O. Box 392, Pretoria, 0003, South Africa

*These authors contributed equally to this work, and are listed in alphabetical order

Contact details:

Daan J.E. Loock: daanje.l@mweb.co.za

Samual T. Williams: samual.t.williams@gmail.com

Kevin W. Emslie: kevin.emslie@gmail.com

Wayne S. Matthews: wayne@wra-africa.net

Lourens H. Swanepoel: lourens.swanepoel.univen@gmail.com

Fig. S1. Cumulative frequency curve showing the relationship between the cumulative number of individual serval identified on the camera traps at Secunda in South Africa.

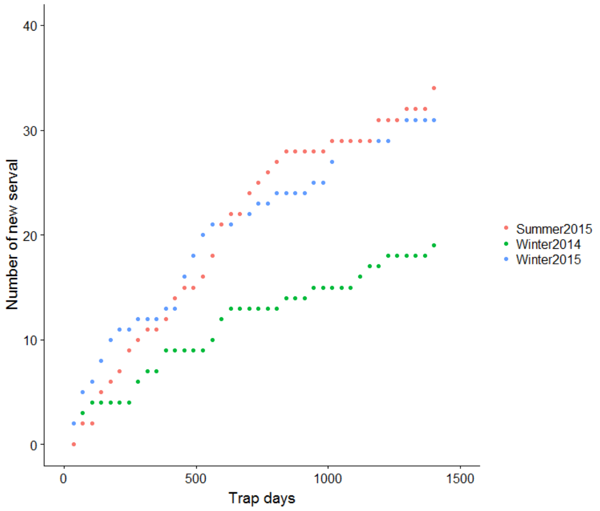

Table S2. Modelling results showing: a) (Mhab.sec) the effect of habitat type on detection probability (g0); b) (Mdens.habitat) the effect of session on density and habitat on detection probability; c) as model b, but allows the scale parameter (sigma) to vary with session; d) (Mb.sec) the effect of behavioural response on detection probability; e) (Mseason) the effect of density affected by season and year; and f) (m0.sec) the null model.

	model	detectfn	npar	logLik	AIC	AICc	dAICc	AICcwt
Mhab.sec	D~1 g0~Habitat	hazard rate	7	-1015.426872	2044.85	2046.33	0.00	0.47

	sigma~1 z~1							
Mdens.habitat	D~session g0~Habitat sigma~1 z~1	hazard rate	9	-1012.996398	2043.99	2046.43	0.10	0.45
Mdens.habitat.ses	D~session g0~Habitat sigma~session z~1	hazard rate	11	-1012.124715	2046.25	2049.92	3.59	0.08
Mb.sec	D~1 g0~b sigma~1 z~1	hazard rate	5	-1028.514939	2067.03	2067.80	21.47	0.00
Mseason	D~session g0~1 sigma~1 z~1	hazard rate	6	-1030.089274	2072.18	2073.27	26.94	0.00
M0.sec	D~1 g0~1 sigma~1 z~1	hazard rate	4	-1033.592632	2075.19	2075.69	29.37	0.00
